# Clinical outcomes of bicuspid versus tricuspid aortic valve stenosis after transcatheter aortic valve replacement with self-expandable valves

**DOI:** 10.1186/s12872-022-02943-9

**Published:** 2022-12-12

**Authors:** Qinchun Jin, Shasha Chen, Xue Yang, Mingfei Li, Wei Li, Xiaochun Zhang, Daxin Zhou, Yat-Yin Lam, Junbo Ge

**Affiliations:** 1 Department of Cardiology, Zhongshan Hospital, Shanghai Institute of Cardiovascular Disease, Fudan University, 180 Fenglin Road, 200032 Shanghai, China; 2 Department of Radiology, Zhongshan Hospital, Fudan University, Shanghai, China; 3 Department of Echocardiology, Zhongshan Hospital, Fudan University, Shanghai, China; 4 Division of Cardiology, Department of Medicine and Therapeutics, Prince of Wales Hospital, Chinese University of Hong Kong, Hong Kong, SAR China

**Keywords:** Bicuspid, Tricuspid, Transcatheter aortic valve replacement, Calcification

## Abstract

**Background:**

There is a lack of available data on specific prognostic comparisons between transcatheter aortic valve replacement (TAVR) using self-expandable valves (SEV) in patients with stenotic Type 0, Type 1 bicuspid aortic valve (BAV) and tricuspid aortic valve (TAV).

**Objectives:**

To evaluate the association between aortic valve morphology and outcomes following self-expandable TAVR.

**Methods:**

Consecutive patients with aortic stenosis(AS) undergoing self-expandable TAVR were enrolled and categorized into three groups (Type 0/Type 1 BAV or TAV) according to the Sievers classification. The primary endpoint was a composite of all-cause mortality and rehospitalization for heart failure (HF) within 2 years. Secondary outcomes included procedural complications and major cardiovascular events observed in clinical follow-ups. Clinical outcomes at 2 years following TAVR were compared among three groups using Kaplan-Meier curve and multivariable Cox proportional hazards regression models.

**Results:**

A total of 344 AS patients (Type 0: 86; Type 1: 109; TAV: 149) were enrolled. The presence of moderate or severe paravalvular leak (PVL) was significantly higher in patients with Type 0 and Type 1 BAV versus TAV (10.47% vs. 16.51% vs. 6.71%, *p* = 0.043). All-cause 30-day mortality (2.33% vs. 0.92% vs. 2.68%, *p* = 0.626) and 2-year mortality (3.49% vs. 5.50% vs. 6.71%, *p* = 0.657) was comparable among the three groups. However, rehospitalization for HF within 2 years was significantly higher in Type 1 BAV (11.63% vs. 20.18% vs. 8.72%, *p* = 0.020). Multivariate Cox analysis showed that a higher STS score, Type 1 BAV morphology and excess leaflet calcification (≥ median calcium volume (CV) of the entire population) were independent predictors for HF rehospitalization. Additional intragroup Kaplan‒Meier analysis showed that excess leaflet calcification could predict higher long-term mortality and rehospitalization risk for HF(HR (95% CI): 3.430 (1.166–10.090), log rank *p* = 0.017) in Type 1 BAV patients.

**Conclusion:**

Outcomes of self-expandable TAVR in BAV-AS patients might vary depending on valve subtypes. BAV patients with excess leaflet calcification and a raphe, especially calcified, had an increased risk of moderate PVL and HF readmission in mid-to-long term follow-ups.

**Supplementary Information:**

The online version contains supplementary material available at 10.1186/s12872-022-02943-9.

## Introduction

BAV is a frequently encountered cardiac malformation that can be divided into three predefined morphological subtypes based on the Sievers classification system [1]. Although BAV has been typically excluded from major large, randomized controlled trials (RCTs) of TAVR due to its challenging anatomical features, recent observational studies and meta-analyses have shown acceptable outcomes of TAVR in BAV-AS patients [2–4]. Whereas, increased risk of paravalvular leak (PVL), conduction abnormalities, valve malposition and annulus rupture in BAV were still reported, and their long-term prognoses remained controversial [5, 6]. Inner-group analysis in BAV-AS patients showed that subjects with a calcified raphe (Type 1) and excess leaflet calcification have less favorable outcomes compared to those with 1 or none of these morphological features [3, 7]. Yet, few reports have investigated TAVR outcomes and complications within specific different aortic valve subgroups, especially in cases of SEV.

The CHOICE Randomized Clinical Trial [8], a study exclusively enrolling TAV patients with second generation THV systems, showed that use of SEVs resulted in lower device success, including higher frequency of moderate PVL, need for a second valve and a new permanent pacemaker implantation (PPM) in comparison to balloon-expandable valves(BEVs). Similar findings were also reported in several recent meta-analyses [9, 10]. The dependence of anchoring and formation on radial force itself makes SEVs more susceptible to the native aortic root structure than BEVs.

Therefore, in this study, we aim to assess the procedural and clinical outcomes between AS patients with Type 0, Type 1 BAV and TAV undergoing TAVR identically using SEVs.

## Methods

### Study design and patient population

**Fig. 1:**
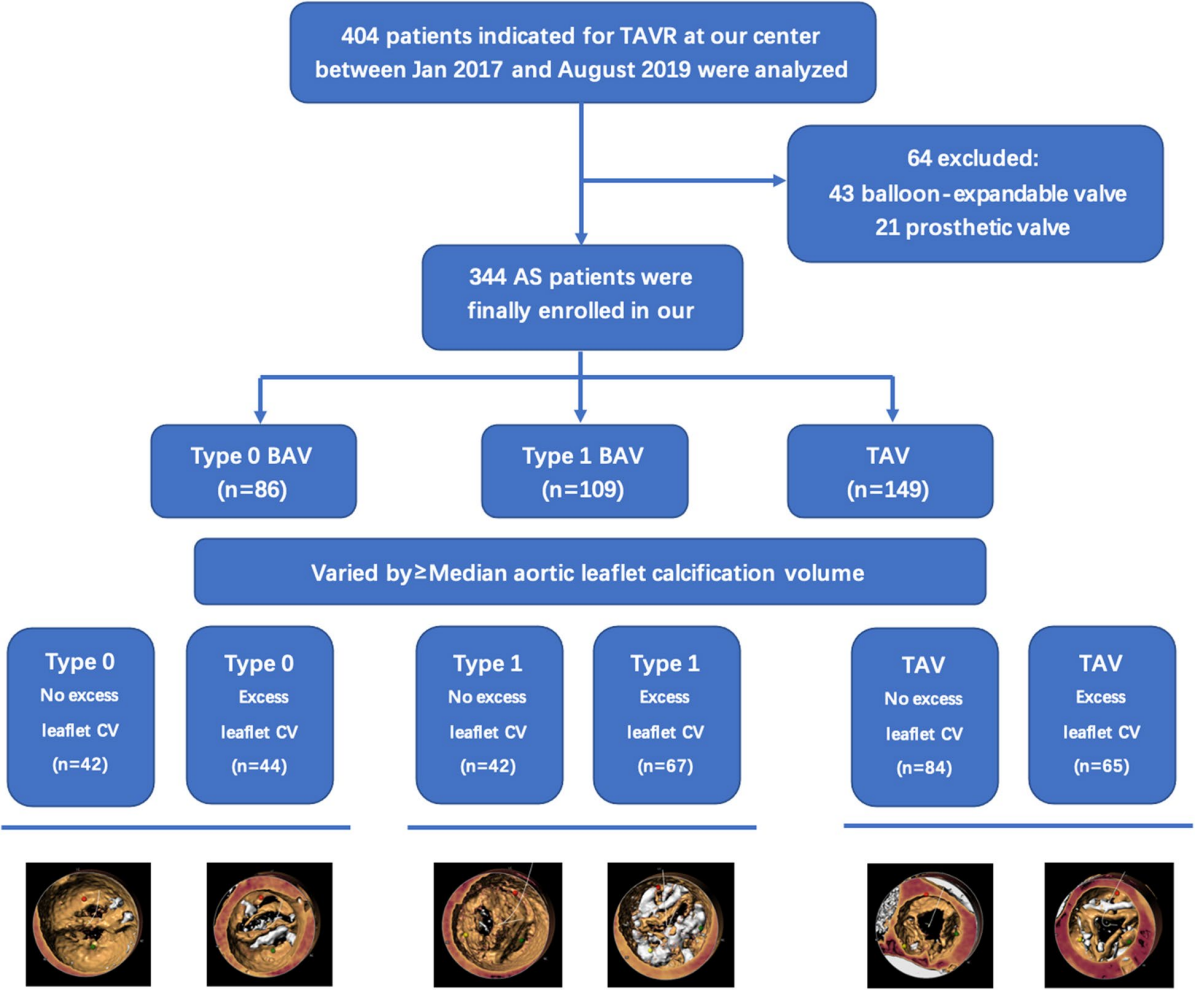
Flow-chart of patient selection. TAVR: transcatheter aortic valve replacement; AS: aortic stenosis; BAV: bicuspid aortic valve; TAV: tricuspid aortic valve; CV: calcium volume

From Jan 2017 to August 2019, consecutive patients with calcific aortic stenosis indicated for self-expandable TAVR in our center were included in this retrospective study (FLOW chart, Fig. 1). Inclusion criteria based on echocardiogram were defined as aortic valve area ≤ 1.0 cm^2^, peak velocity ≥ 4 m/s or a mean transvalvular gradient ≥ 40 mmHg. Multidetector computed tomography (MDCT) was used for procedural planning.

TAVR procedures were conducted under general anesthesia and under guidance of transesophageal echocardiography (TEE) as previously described [11]. Informed consent was obtained from each subject, and our study was approved by the Institutional Review Board of Zhongshan Hospital, Fudan University, Shanghai, China.

### Multidetector computed tomography and sizing strategy

All patients were evaluated by an ECG-gated, multi-slice CT angiography study with a 320-detector row computed tomography scanner (Aquilion One, Canon Medical Systems, Tochigi-ken, Japan). Reconstructions of the aortic root were created using 3mensio software (Pie Medical Imaging, Bilthoven, the Netherlands) as previously described [12] and the slide thickness was 0.5 mm. All CT images were independently reviewed by two experienced cardiologists for interobserver agreement at our institution using reliable TAVR planning software (3mensio Structural Heart, 3mensio Medical Imaging B.V., Bilthoven, the Netherlands). After CT assessment, patients were classified according to the Sievers classification system as follows: BAV (type 0: congenitally malformed valve of 2 symmetric cusps without a raphe; types 1 and 2 were diagnosed when 1 or 2 raphes were presented) and TAV [1]. The quantity and distribution of calcification were analyzed by using calcium volume (CV) measurements with an empirical starting threshold of 850 HU according to the contrast-enhanced images [13]. Aortic valve calcification was separated into two regions along the double oblique long-axis of the left ventricular outflow tract (LVOT) and the aortic annulus: LVOT (from the basal annular plane to 5 mm below the left ventricle) and aortic valve leaflets (from the annular plane to each cuspid tip) [14] (Supplementary Fig. 1). Leaflets with a CV greater than the median value in the analyzed cohort were further categorized as having excess leaflet calcification. For LVOT calcification, moderate/severe calcification of the LVOT was determined as the presence of one nodule of calcification extending ≥ 5 mm and covering ≥ 10% of the perimeter of the LVOT, as previously described [15].

Our selection strategy for valve size was commonly based on the annulus dimensions of the individual image reconstructions in the end-systolic phase (35% of the cardiac cycle) while the nominal thresholds for each size were derived from vendor recommendations. Undersizing (defined as a smaller prosthesis as opposed to the preprocedural CT-predicted size) would be considered in case of those with severe calcification in the sinus of Valsalva, LVOT and commissure fusion, and evaluated at high risk of coronary obstruction and pacemaker implantation.

### Outcomes and definitions

The primary endpoint of our present study was the combination of all-cause mortality and rehospitalization for heart failure (HF) (defined as any event requiring oral and/or intravenous therapy) at 2 years following the TAVR procedure. Secondary outcomes were major clinical endpoints (including stroke, major bleeding, valve-related reintervention, percutaneous coronary artery intervention (PCI), vascular complications, new permanent pacemaker implantation (PPM) and valve thrombosis) based on the Valve Academic Research Consortium 3 (VARC3) criteria [16] during follow-up.

### Data collection

Baseline clinical, laboratory and procedural data were collected. Echocardiographic parameters and MDCT-derived measurements were obtained as part of routine diagnostic work-up. Routine follow-ups were conducted after discharge for each subject through clinical visits and/or through telephone calls at prespecified intervals (30 days post-TAVR and every 6 months thereafter).

### Statistical analysis

Continuous variables were presented as the mean ± SD or medians (interquartile range [IQR]) as appropriate. Univariate ANOVA or Kruskal‒Wallis was then used to compare continuous variables between the three groups. Categorical variables were reported as counts (percentages) and compared using the chi-squared test or Fisher’s exact test. Primary outcomes at 2 years were calculated through Kaplan‒Meier survival analysis and compared using the log-rank test. Univariate Cox regression models were used to identify predictors of poor clinical outcomes during follow-ups, and variables with p < 0.1 were considered eligible for inclusion in the multivariate Cox analysis. To further determine the prognostic effects of aortic valve calcification in each group, receiver operating characteristic (ROC) curves were generated according to different defined endpoints. The area under the curve (AUC) was calculated. Cutoff values from the ROC analysis with the highest combination of sensitivity and specificity were chosen.

All statistical analyses were performed using SPSS software version 26.0. A two-sided *p* value of < 0.05 was considered statistically significant.

## Result

### Baseline characteristics

A total of 404 AS patients undergoing TAVR were identified at our center during this period. Subjects with missing data, degenerative bioprostheses, and those receiving BEVs were excluded from our analysis. Consequently, 344 patients were finally enrolled in our study and divided into three groups (Type 0 BAV: 86; Type 1 BAV: 109; TAV: 149) based on their valve morphology (Fig. 1).

All baseline demographics were summarized in Table 1. While most characteristics were well balanced between the three groups, patients with Type 0 or Type 1 BAV were significantly younger than TAV(72.90 ± 7.06 vs. 76.31 ± 7.17 vs. 77.05 ± 9.32, *p* = 0.001). A higher proportion of hypertension (37.21% vs. 52.29% vs. 74.50%, *p* < 0.001) and higher Society of Thoracic Surgeons (STS) predicted risk of mortality score (4.69 ± 1.99 vs. 4.50 ± 1.59 vs. 5.14 ± 2.15, *p* = 0.025) were observed in patients with TAV.

**Table 1:** Baseline characteristics

	TYPE0 BAV (*n* = 86)	TYPE1 BAV (*n* = 109)	TAV (*n* = 149)	*P* value
Age(yrs)	72.90 ± 7.06	76.31 ± 7.17	77.05 ± 9.32	0.001
Sex, male(n.%)	41 (47.67)	55 (50.46)	77 (51.68)	0.839
BMI	23.86 ± 2.84	23.05 ± 2.93	23.96 ± 3.75	0.085
NYHA ≥ III(n.%)	68 (79.07)	91 (83.49)	122 (81.88)	0.729
STS score	4.15 ± 1.79	4.50 ± 1.59	5.14 ± 2.15	< 0.001
Hypertension(n.%)	32 (37.21)	57 (52.29)	111 (74.50)	< 0.001
Diabetes mellitus (n.%)	18 (20.93)	18 (16.51)	43 (28.86)	0.058
Creatine(mg/dL)	92.52 ± 91.05	96.22 ± 49.26	119.17 ± 121.68	0.068
Peripheral vascular disease(n.%)	6 (6.98)	16 (14.68)	25 (16.78)	0.101
Prior cerebrovascular(n.%) accident	6 (6.98)	8 (7.34)	11 (7.38)	0.914
Chronic lung disease(n.%)	45 (52.33)	62 (56.88)	79 (53.02)	0.772
Cardiac history(n.%)				
PCI	6 (6.98)	8 (7.34)	20 (13.42)	0.157
CABG	1 (1.16)	1 (0.92)	5 (3.36)	0.383
AF/Af	21 (24.42)	22 (20.18)	27 (18.12)	0.526
Pre-existing pacemaker	2 (2.33)	4 (3.67)	3 (2.01)	0.696
Echocardiographic findings				
Mean gradient(mmHg)	49.06 ± 20.81	45.37 ± 17.15	44.39 ± 15.42	0.152
Aortic valve area(mm²)	0.62 ± 0.12	0.74 ± 0.43	0.69 ± 0.15	0.073
LVEF(%)	56.26 ± 15.51	55.83 ± 12.98	55.44 ± 15.71	0.920
Maximum aortic valve Velocity(m/s)	4.90 ± 0.81	4.77 ± 0.72	4.65 ± 0.76	0.528
Moderate-to-severe aortic regurgitation (n.%)	4 (4.65)	4 (3.67)	14 (9.40)	0.161
Moderate-to-se vere mitral regurgitation	2 (2.33)	2 (1.83)	9( 6.04)	0.207
Pre-CT data				
Annulus				
Area(mm²)	467 (419–548)	496(431–567)	441(377–490)	0.475
Perimeter(mm)	80.30 ± 7.01	81.98 ± 8.35	80.61 ± 7.67	0.336
RCA height(mm)	15.88 ± 3.61	14.97 ± 3.73	14.84 ± 3.07	0.584
LM height(mm)	17.35 ± 3.59	15.44 ± 3.20	15.92 ± 3.46	0.115
Total leaflet CV(mm³)	511 (287.73-885.53)	622.50 (359.70-915.80)	417 (217–714.00)	< 0.001
Total LVOT CV(mm³)	0 (0-34.28)	0 (0-33.05)	0 (0-28.93)	0.547
Excess leaflet calcification(n,%)	45 (52.33)	68 (62.39)	57 (38.26)	
Moderate to severe LVOT calcification(n,%)	27 (31.40)	30 (27.52)	41 (27.52)	0.878

*BAV* bicuspid aortic valve, *BMI *body mass index, *NYHA *New York Heart Association, *STS *Society of Thoracic Surgeons, *PCI *percutaneous coronary artery intervention, *AF *atrial fibrillation, *Af *atrial flutter, *CABG *Coronary artery bypass grafting, *LVEF* left ventricular ejection fraction, *CT* computed tomography, *RCA* right coronary artery, *LM* left main, *LVOT* left ventricular outflow tract, *CV* calcium volume

In terms of pre-TAVR echocardiographic measurements, left ventricular ejection fraction (LVEF), mean transvalvular gradient, aortic valve area, and maximum aortic valve velocity were similar among the three groups. Total leaflet CV was significantly higher (511 (287.73-885.53) mm³ vs. 622.50 (359.70-915.80) mm³ vs. 417 (217.75–714.00) mm³) in Type 0, Type 1 BAV than TAV (*p* < 0.001).

### Procedural characteristics and in-hospital outcomes:

The procedural and in-hospital outcomes of our study population are shown in Table 2. There was no significant difference in vascular access route or type of implanted valve among the three groups. Moreover, downsizing and post-dilation were more commonly performed for patients with Type 0 and Type 1 BAV stenosis (33.72% vs. 31.10% vs. 15.44%, *p* = 0.001; 41.86% vs. 33.94% vs. 22.82%, *p* = 0.007). With regard to valve hemodynamic findings, mean transvalvular gradients were significantly higher in Type 0 and Type 1 BAV(12.76 ± 6.13 mmHg vs. 12.25 ± 6.09 mmHg vs. 10.36 ± 4.84 mmHg, *p* = 0.005) following self-expandable TAVR in comparison to TAV. Among these three groups, patients with Type 1 BAV stenosis had a significantly higher rate of ≥ moderate paravalvular leakage (9.30% vs. 21.10% vs. 12.08%, *p* = 0.039). There were no significant differences in in-hospital events, such as all-cause mortality, stroke, new pacemaker implantation, major bleeding, vascular complications or acute kidney injury.

**Table 2:** Procedural and clinical outcomes

Procedural outcomes	TYPE 0 BAV (*n* = 86)	TYPE 1 BAV (*n* = 109)	TAV (*n* = 149)	*P* value
Transfemoral access(n,%)	86 (100.00)	105 (96.33)	144 (96.64)	0.204
Device(n,%)				
Venus-A	53 (60.63)	59 (54.13)	89 (59.73)	0.523
Vita-flow	33 (38.37)	50 (45.87)	60 (40.27)	
Down sizing(n,%)	29 (33.72)	34 (31.10)	23 (15.44)	0.001
Procedural outcomes				
Post-dilation(n,%)	36 (41.86)	37 (33.94)	34 (22.82)	**0.007**
Depth(mm)	4.54 ± 3.27	4.31 ± 3.70	4.63 ± 3.05	0.794
Conversion to open surgery(n,%)	2 (2.33)	1 (0.92)	2 (1.34)	0.727
Coronary obstruction(n,%)	0 (0)	0 (0)	1 (0.67)	≥ 0.999
Aortic root injury(n,%)	0 (0)	0 (0)	1 (0.67)	≥ 0.999
Implantation of two valves(n,%)	6 (6.98)	9 (8.26)	8 (5.37)	0.651
Echocardiographic findings				
Mean gradient(mmHg)	12.76 ± 6.13	12.25 ± 6.09	10.36 ± 4.84	**0.005**
≥ moderate pvl(n,%)	9 (10.47)	18 (16.51)	10 (6.71)	**0.043**
LVEF(%)	61.66 ± 8.44	62.13 ± 7.42	62.13 ± 7.42	0.425
In-hospital events(n,%)				
All-cause mortality	2 (2.33)	1 (0.92)	3 (2.01)	0.768
Stroke	3 (3.49)	5 (4.59)	2 (1.34)	0.273
New permanent pacemaker	9 (10.47)	16 (14.68)	22 (14.77)	0.608
Major bleeding	0	2 (1.83)	2 (1.34)	0.688
Major vascular complications	2 (2.33)	1 (0.92)	3 (2.01)	0.768
Acute kidney injury	1 (1.16)	0 (0)	1 (0.67)	0.768

### Clinical outcomes during follow-up

**Fig. 2:**
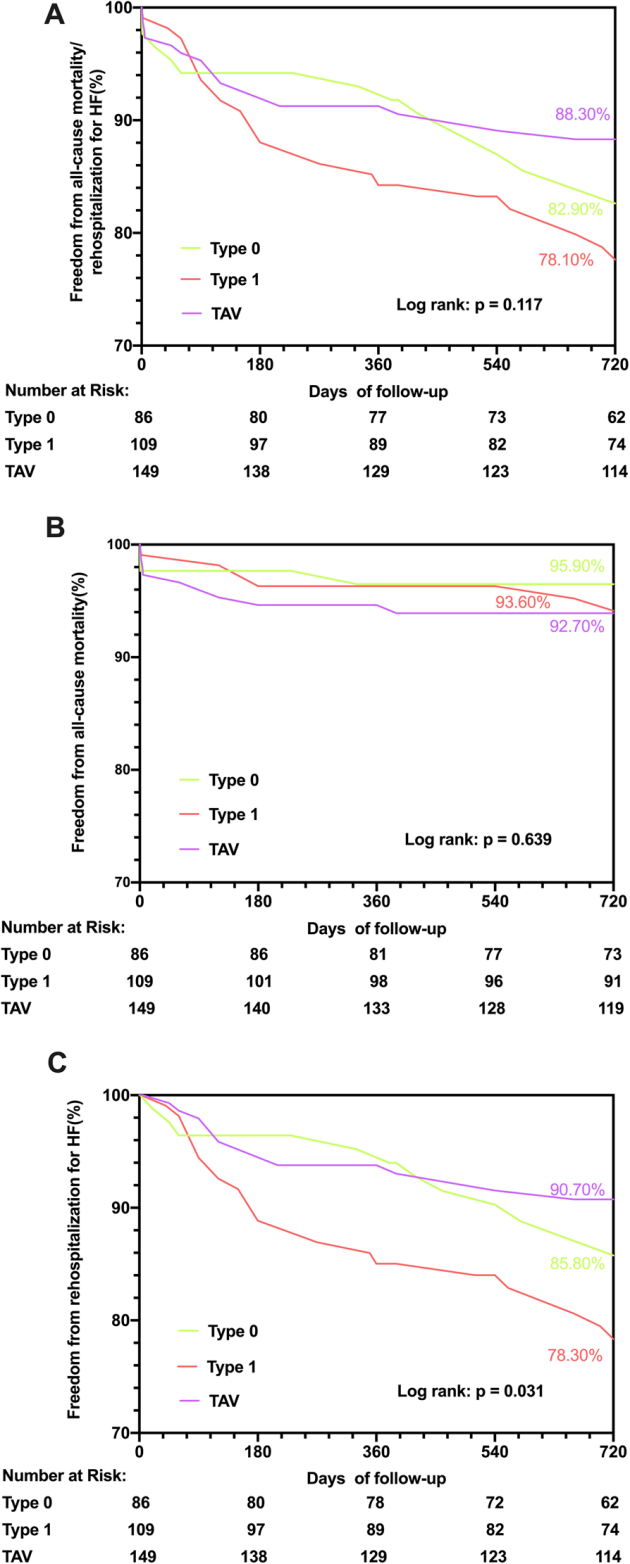
Kaplan-Meier analysis for the primary endpoint between three groups. **A** All-cause mortality and rehospitalization for heart failure; **B **All-cause mortality; **C **Rehospitalization for heart failure

In our study population, a total of 10/86 (11.63%) patients with Type 0 BAV, 12/109 (11.01%) patients with Type 1 BAV and 15/149 (10.07%) patients with TAV were lost to follow-up during two years after discharge. At 2 years, the rate of freedom from the composite of all-cause mortality and rehospitalization for HF was not significantly different (82.90% vs. 78.10% vs. 88.30%, log rank *p* = 0.117) among the three groups (Fig. 2A). The secondary endpoint results at 30 days, 1 year and 2 years following TAVR were listed in Table 3. There was no significant difference among these three groups in terms of the occurrence of all-cause mortality (Fig. 2B), stroke, myocardial infarction, coronary intervention, major bleeding, valve thrombosis, new pacemaker implantation or need for valve reintervention. However, lower rates of freedom from rehospitalization for HF were reported in the Type 1 BAV group (85.80% vs. 78.30% vs. 90.70%, log rank *p* = 0.031) (Fig. 2C). In the multivariable analysis, factors independently associated with 2-year rehospitalization for HF were STS score (HR: 1.386, 95% CI: 1.222–1.572, *p* < 0.001), Type 1 BAV phenotype (HR: 3.034, 95% CI: 1.435–6.415, *p* = 0.004) and excess leaflet calcification (HR: 3.304, 95% CI: 1.615–6.762, *p* = 0.001) (Table 4).

**Table 3:** Clinical outcomes during follow-up

	30 days				1 year				2-year			
	Type 0 BAV	Type 1 BAV	TAV	*P* value	Type 0 BAV	Type 1 BAV	TAV	*P* value	Type 0 BAV	Type 1 BAV	TAV	*P* value
All cause mortality (n,%)	2 (2.33)	1 (0.92)	4 (2.68)	0.626	3 (3.49)	4 (3.67)	8 (5.37)	0.794	3 (3.49)	6 (5.50)	9 (6.04)	0.782
Stroke (n,%)	4 (4.65)	6 (5.50)	3 (2.01)	0.278	4 (4.65)	8 (7.34)	5 (3.36)	0.315	4 (4.65)	8 (7.34)	6 (4.03)	0.499
MI (n,%)	0	0	0	-	0	0	0	-	0	0	0	-
Major Bleeding (n,%)	0	2 (1.83)	2 (1.34)	0.688	1 (1.16)	3 (2.75)	3 (2.01)	0.797	1 (1.16)	3 (2.75)	4 (2.68)	0.813
Valve thrombosis (n,%)	0	0	0	-	0	1 (0.92)	0	0.567	0	1 (0.92)	0	0.567
Pacemaker (n,%)	7 (8.14)	15 (13.76)	22 (14.77)	0.323	9 (8.26)	15 (13.76)	22 (14.77)	0.628	10 (11.63)	17 (15.60)	22 (14.77)	0.739
PCI (n,%)	0	0	0	-	1 (1.16)	0	1 (0.67	0.725	2 (2.33)	0	1 (0.67)	0.342
Reintervention (n,%)	0	0	0	-	0	0	1 (0.67)	≥ 0.999	0	1 (0.92)	1 (0.67)	≥ 0.999
Rehospitalization for HF (n,%)	2 (2.33)	0	0	0.062	4 (4.65)	16 (14.68)	9 (6.04)	0.016	11 (12.79)	22 (20.18)	13 (8.72)	**0.020**

*MI* myocardial infarction, *HF *heart failure, *PCI *percutaneous coronary artery intervention

**Table 4:** Independent predictor of rehospitalization for HF among the entire study group

	Univariate model		Multivariate model	
	HR (95%CI)	*P* value	HR (95%CI)	*P* value
Age, per increase of 1 yr	1.009 (0.969–1.051)	0.660	-	-
Male	0.388 (0.193–0.783)	0.004	-	-
Sts score	1.305 (1.170–1.455)	< 0.001	1.386 (1.222–1.572)	< 0.001
BMI	0.995 (0.908–1.091)	0.922	-	-
NYHA functional class III or IV	1.166 (0.544–2.502)	0.688	-	-
Down Sizing	1.947 (1.070–3.544)	0.029	-	-
Hypertension	0.905 (0.507–1.617)	0.737	-	-
AF	1.872 (0.999–3.509)	0.050	-	-
Valve Morphology (TAV as control)	-	0.035	-	0.015
Type0	1.395 (0.625–3.114)	0.417	1.994 (0.872-4.561)	0.102
Type1	2.406 (1.212–4.776)	0.012	3.034 (1.435–6.415)	0.004
Excess leaflet calcification	3.460 (1.757–6.814)	< 0.001	3.304 (1.615–6.762)	0.001
Moderate to severe LVOT calcification	2.385 (1.306–4.356)	0.005	-	-

Abbreviations as above

### Impacts of aortic valve calcification on clinical outcomes in each valve phenotype:

**Fig. 3:**
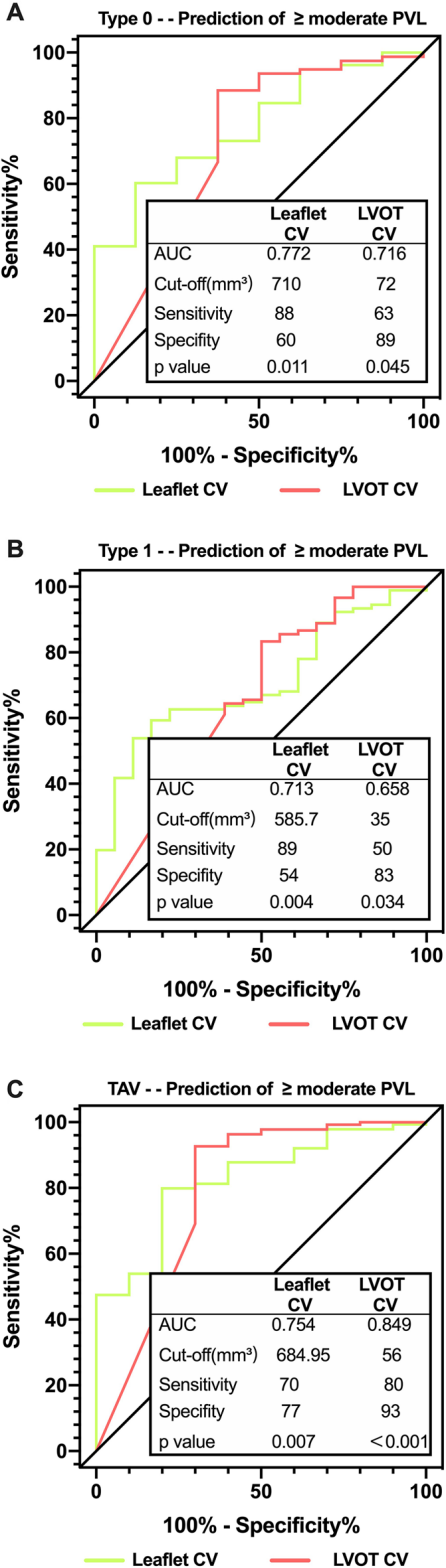
ROC analysis for prediction of significant PVL for different valve types. Type 0 BAV; （**B**）Type 1 BAV; (**C**) TAV. PVL: paravalvular leak; BAV: bicuspid aortic valve; (**C**) tricuspid aortic valve. ROC: receiver operating characteristic; PVL: paravalvular leak

Considering the significant difference of AVC among three groups at baseline, the binary classification merely based on ≥ median CV of the entire study population might be insufficient to draw conclusion on the impacts of AVC on clinical outcomes following TAVR. Hence, further intragroup evaluation of clinical outcomes varied by excess leaflet calcification((≥ median CV of the specific study population)) in each valve phenotype were performed(Supplementary Tables 1– 3). Regarding postprocedural endpoints, the rates of moderate/severe PVL were both significantly higher among patients with severe leaflet calcification (Supplementary Fig. 2). ROC curves showed that both aortic valve leaflet and LVOT CV were positive predictors for significant PVL regardless of valve phenotype. However, in comparison with LVOT CV, a significantly stronger association of leaflet CV prediction could be observed in patients with Type 0 (AUC: 0.814 vs. 0.651) and Type 1 BAV (AUC: 0.682 vs. 0.621) but not in TAV (Fig. 3).

**Fig. 4:**
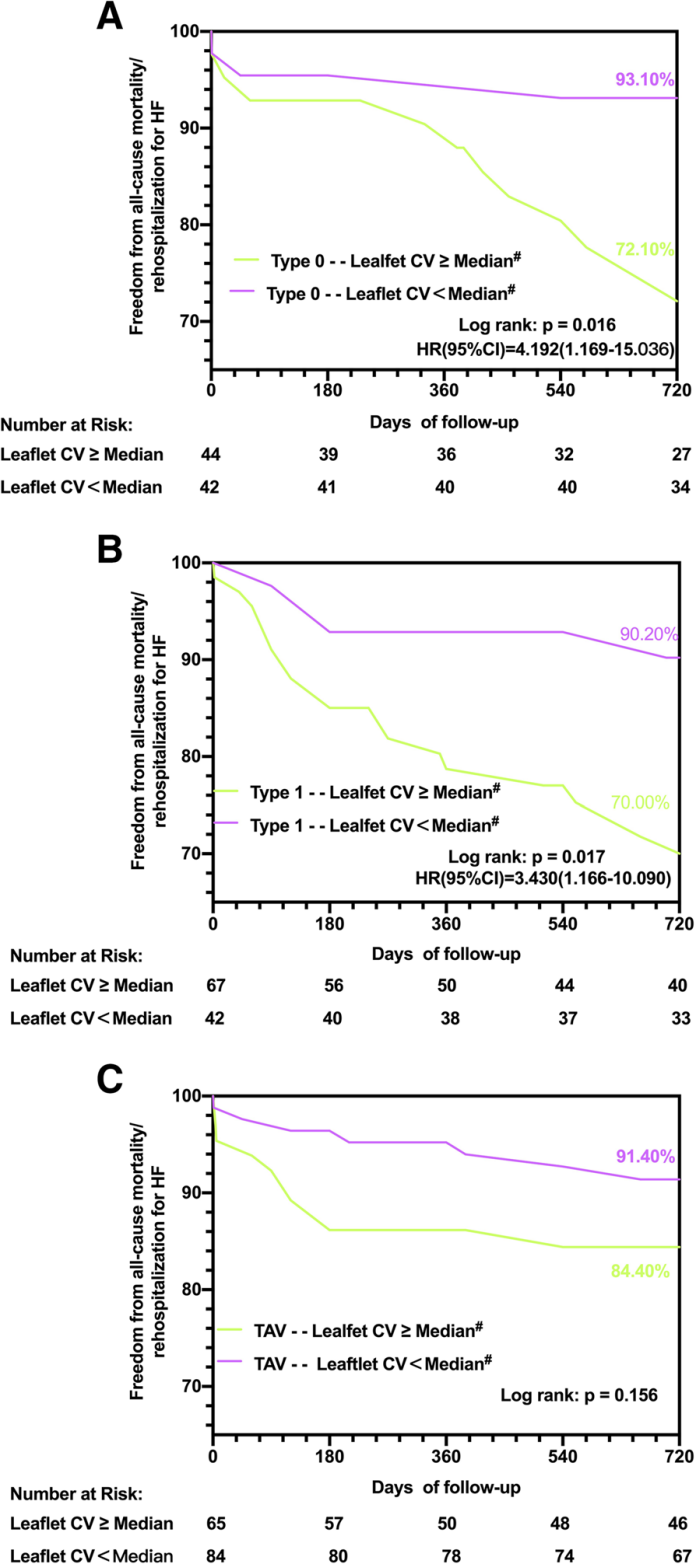
Kaplan-Meier estimates of the rate of the primary composite endpoint in different aortic valve phenotype as varied by leaflet CV ≥ median among the entire population

Intragroup Kaplan‒Meier analysis was performed and varied by excess leaflet CV (≥ median CV of the entire study population) (Fig. 4) in each subtype. For patients with Type 0 (93.10% vs. 72.10%, log rank *p* = 0.016, HR (95% CI): 3.192 (1.169–15.036)) and Type 1 BAV (90.20% vs. 70.00% log rank *p* = 0.017, HR (95% CI): 3.430 (1.166–10.090)), excess leaflet calcification was an independent predictor for all-cause mortality and rehospitalization for HF in the 2-year follow-up, but no significant difference was shown in TAV(91.40% vs. 84.40, log rank *p* = 0.156). Considering the degree of CV dispersion within each group, we performed an additional Kaplan‒Meier analysis according to the intragroup excess leaflet CV (≥ median CV of the specific study population) (Supplementary Fig. 2) in each subtype. Patients with Type 1 BAV (87.00% vs. 68.70%, log rank *p* = 0.033, HR (95% CI): 2.525 (1.038–6.139)) were still at higher risk of poor clinical outcomes after self-expandable TAVR if they presented with an excessive leaflet CV, but both Type 0 BAV(90.20% vs. 75.50%, log rank *p* = 0.082) and TAV patients(87.80% vs. 88.90%, log rank *p* = 0.889) failed (Supplementary Fig. 3).

## Discussion

In recent years, the efficacy of TAVR in BAV patients has drawn attention worldwide. Despite increased operator experience and advances in prosthetic design, outcomes of TAVR with SEVs in BAV-AS patients remain inconsistent. In the present study, we demonstrated that patients with Type 1 BAV were at higher risk of paravalvular leak post-TAVR and had a poor prognosis in the mid-to-long term follow-up, especially those with excessive aortic leaflet calcification. Therefore, individual anatomical characteristics including aortic valve phenotype and calcification should be carefully evaluated for TAVR planning in BAV patients. Optimal patient selection based on CT assessments could be of great importance to further improve the prognosis of TAVR in BAV-AS population.

### Periprocedural complications

Given the limited data for morphological subtypes in previous studies exploring the feasibility of TAVR in BAV in comparison to TAV, we divided our study population into three groups (Type 0/ Type 1 BAV and TAV) according to the commonly used Sievers classification [1]. In our study, BAV-AS patients showed comparable rates of periprocedural death, risk of PPM and other cardiovascular complications but a higher prevalence of PVL and transvalvular gradient than TAV patients, as addressed in other observational studies [17]. The higher risk of PVL in patients with Type 1 BAV is further highlighted in our findings. The bulky leaflet calcification, commissural fusion and calcified raphe in Type 1 BAV could significantly limit the prosthetic expansion [18] and result in suboptimal valve performance [19]. In the case of SEV, a prosthetic type more susceptible to landing zone calcium mass [20, 21], that effect might be exaggerated. Hence, to some extent, based on our findings, we acknowledge the need to clearly elucidate the varied proportion of BAV subtypes in studies to reduce selection bias. Regarding stroke events, although there was no significant difference, two- and threefold higher rates were observed in BAV patients than in TAV patients. Hahn RT et al. [22] reported that post-dilation could contribute to early stroke, and higher rates of disabling stroke under a heavier burden of aortic valvular complex calcification as documented by Euihong Ko [23]. Hence, despite the overall low stroke rates presented in our study, it would be worthwhile to thoroughly investigate the predictors of stroke in a larger sample of BAV patients. Nevertheless, it must be said that a significantly higher ratio of BAV patients was presented in our study. It could be attributed to the our single center study design and regional differences in China [24–27]. Hence, investigations based on a multi-center database or a propensity-score matched cohort are warranted to provide further evidence for our findings.

### Survival and rehospitalization for HF during follow-up

For clinical follow-ups, the present study showed no difference in all-cause mortality at 30 days and 2 years after discharge between the three groups, as has also been shown in previous studies [2, 28]. While risk factors such as advanced age, higher STS score and more baseline comorbidities were more prevalent in TAV patients, the incidence of rehospitalization for HF, another important performance indicator, was significantly higher in Type 1 BAV patients, followed by Type 0 BAV and TAV (20.18% vs. 11.63% vs. 8.72%, *P* = 0.020) groups, according to our analysis. This difference may be explained by the greater risk of moderate PVL in Type 1 BAV subgroup. This continuous overloaded regurgitation volume has been identified as a positive predictor for cardiac function deterioration post-AVR [29]. Our findings support the concern of poor prognosis in BAV patients with fused calcified raphe as raised by Yoon SH et al. [7] and have further underlined the inferiority of this comparison to TAV. Thus, focusing on the specific differentiation of bicuspid morphology and anatomy upon a larger database may facilitate the evaluation of the long-term prognosis of BAV and TAV AS patients undergoing TAVR using SEV.

### Leaflet calcification on outcomes

Valvular calcification, one of the most common additional anatomical abnormalities coexisting with BAV, is believed to be an important issue in TAVR planning. Our study also showed that excessive calcification was associated with worse PVL and poor follow-up outcomes post-TAVR. However, considering the difference in CV burden among the three groups at baseline, we also performed an intragroup analysis to specify the impact of device landing zone calcium on each valve morphology. The positive association between leaflet as well as LVOT calcification and post-TAVR PVL incidence shown in our study has also been reported in previous studies [12, 30]. Moreover, we have identified a stronger relationship between the prediction of PVL by leaflet CV in BAV subgroups. One possible contributor is the supra annular sizing strategy for BAVs applied in our center [31], which usually results in a higher implantation level of TAVR prostheses than the standard annulus-based selections [32]. Therefore, SEVs could be highly susceptible to the surrounding aortic cuspid calcium burden but less influenced by LVOT calcification. A notable potential pitfall to address in our study is that the sensitivity and specificity of leaflet calcification and PVL in BAV patients upon ROC analysis was less acceptable, which might be related to our small study sample size and the interobserver bias. In terms of long-term prognosis, our intragroup analysis showed that an excessive leaflet CV was indeed highly associated with the risk of all-cause mortality and rehospitalization for HF in Type 1 BAV (HR (95% CI): 2.525 (1.038–6.139), *p* = 0.033), and an adverse trend could also be observed in Type 0 BAV (*p* = 0.082). Nevertheless, no significant relationship was observed in the TAV. Considering the strength of the relationship between leaflet CV and significant PVL in the BAV group, we speculated that the non-circular landing zone, especially zones with a raphe (normally calcified), might maintain elliptic distortion or noncircular expansion of the implanted frame, further significantly limiting space for continuous outward expansion of SEV to regress PVL, as previously described [33]. Overall, our findings indicated that variations in the proportion of aortic valve subtypes, calcification status, prosthesis type were possible explanations for the conflicting results of outcomes between patients with BAV and TAV patients following TAVR documented in different studies. The present study appears to be a strong wake-up call for better patient selection when performing TAVR in AS patients with degenerative bicuspid anatomy, especially in Type 1 BAV patients with severe calcified leaflet and raphe.

### Limitations

This study was limited in several ways. First of all, it was a retrospective, single-center study design, and the unmeasured confounders might have influenced the present findings. Secondly, the related imaging data and clinical events were not adjudicated by an independent core laboratory. Thirdly, given the limited sample size of patients and clinical events during the 2-year follow-ups, our study might be underpowered to detect the real difference in complication occurrence in different valve morphologies according to the volume of calcium. Therefore, the overall findings of our present analysis should be considered exploratory and hypothesis-generating only.

## Conclusion

Outcomes of self-expandable TAVR in BAV-AS patients might vary depending on valve subtypes. BAV patients with excess leaflet calcification and a raphe, especially calcified, had an increased risk of moderate PVL and HF readmission in mid- to long-term follow-ups. Our data suggested that although self-expandable TAVR could be indicated for BAV-AS patients, it should still be cautiously selected, considering the anatomical risk assessed by CT in conjunction with surgical risk.

## Supplementary Information

**Additional file 1: Supplementary Table 1.** Procedural and clinical outcomes according to excess leaflet CV in Type 0 BAV. **Supplementary Table 2.** Procedural and clinical outcomes according to excess leaflet CV in Type 1 BAV.** Supplementary Table 3. **Procedural and clinical outcomes according to excess leaflet CV in TAV.

**Additional file 2: Supplementary Figure 1.** Quantitative analysis of aortic valve calcification Two regions are defined: aortic valve leaflet(from the basal annular plane to each cuspid tip(red bracket)); left ventricular outflow tract(from basal annular plane to 5mm underneath the left ventricle(yellow bracket)).

**Additional file 3: Supplementary Figure 2.** Comparisons of aortic valve calcium volume dichotomized by moderate/severe PVL. (A) Total aortic valve Leaflet CV; (B) Total LVOT CV.

**Additional file 4: Supplementary Figure 3.** Kaplan-Meier estimates of the rate of the primary composite endpoint in different aortic valve phenotype as varied by leaflet CV ≥ median among the specific group. (A) Type 0 BAV; (B) Type 1 BAV; (C) TAV.

## Data Availability

The datasets generated and/or analyzed in the current study are not publicly available due to restrictions enforced by national and institutional regulations. Data may be obtained if a reasonable request is made to the corresponding author (zhou.daxin@outlook.com) following approval from the ethics committee.

## Abbreviations

AUC: area under the curve
AS: aortic stenosis
BAV: bicuspid aortic valve
CV: calcium volume
HF: heart failure
IQR: interquartile range
LVEF: left ventricular ejection fraction
LVOT: left ventricular outflow tract
MDCT: multidetector computed tomography
PVL: paravalvular leak
ROC: receiver-operating characteristic
STS: Society of Thoracic Surgeons
TAV: tricuspid aortic valve
TAVR: transcatheter aortic valve replacement
TEE: transesophageal echocardiography
VARC3: Valve Academic Research Consortium 3
