# Correction: Clinical outcomes of bicuspid versus tricuspid aortic valve stenosis after transcatheter aortic valve replacement with self‑expandable valves

**DOI:** 10.1186/s12872-023-03041-0

**Published:** 2023-01-17

**Authors:** Qinchun Jin, Shasha Chen, Xue Yang, Mingfei Li, Wei Li, Xiaochun Zhang, Daxin Zhou, Yat-Yin Lam, Junbo Ge

**Affiliations:** 1 Department of Cardiology, Zhongshan Hospital, Shanghai Institute of Cardiovascular Disease, Fudan University, 180 Fenglin Road, 200032 Shanghai, China; 2 Department of Radiology, Zhongshan Hospital, Fudan University, Shanghai, China; 3 Department of Echocardiology, Zhongshan Hospital, Fudan University, Shanghai, China; 4 Division of Cardiology, Department of Medicine and Therapeutics, Prince of Wales Hospital, Chinese University of Hong Kong, Hong Kong, SAR China

## Correction to: BMC Cardiovascular Disorders (2022) 22:540 https://doi.org/10.1186/s12872-022-02943-9

Following publication of the original article [1], in this article the affiliation details for authors Xiaochun Zhang and Yat-Yin Lam were swapped but it should read as follows:

Qinchun Jin^1†^, Shasha Chen^1†^, Xue Yang^2†^, Mingfei Li^1^, Wei Li^3^, Xiaochun Zhang^1*^, Daxin Zhou^1*^, Yat-Yin Lam^4*^ and Junbo Ge^1^

^1^Department of Cardiology, Zhongshan Hospital, Shanghai Institute of Car‑diovascular Disease, Fudan University, 180 Fenglin Road, 200032 Shanghai, China. ^2^Department of Radiology, Zhongshan Hospital, Fudan University, Shanghai, China. ^3^Department of Echocardiology, Zhongshan Hospital, Fudan University, Shanghai, China. ^4^Division of Cardiology, Department of Medicine and Therapeutics, Prince of Wales Hospital, Chinese University of Hong Kong, Hong Kong, SAR, China.

The original article has been corrected.

## References

[1] Jin et al. BMC Cardiovascular Disorders 10.1186/s12872-022-02943-9

