# Supplementary material for: Clinical outcomes of bicuspid versus tricuspid aortic valve stenosis after transcatheter aortic valve replacement with self-expandable valves
Source: BMC Cardiovasc Disord. 2022 Dec 12;22:540. doi: 10.1186/s12872-022-02943-9 (PMC9743542; doi:10.1186/s12872-022-02943-9)
Supplement: Supplementary file 1 — Additional file 1: Supplementary Table 1. Procedural and clinical outcomes according to excess leaflet CV in Type 0 BAV. Supplementary Table 2. Procedural and clinical outcomes according to excess leaflet CV in Type 1 BAV. Supplementary Table 3. Procedural and clinical outcomes according to excess leaflet CV in TAV. [file 12872_2022_2943_MOESM1_ESM.docx]

Supplementary table 1 Procedural and clinical outcomes according to excess leaflet CV in Type 0 BAV

| Type0 | No excess leaflet calcification(n=42) | Excess leaflet calcification(n=44) | P value |
| --- | --- | --- | --- |
| Procedural related outcomes |  |  |  |
| Conversion to open surgery(n,%) | 1(2.38) | 0 | 0.488 |
| Coronary obstruction(n,%) | 0 | 0 | - |
| Aortic root injury(n,%) | 0 | 0 | - |
| Implantation of two valves(n,%) | 3(7.14) | 2(4.54) | 0.673 |
| Stroke | 0 | 3(6.82) | 0.242 |
| Pacemaker | 7(16.67) | 2(4.54) | 0.085 |
| Echocardiographic findings |  |  |  |
| Mean transvalvular gradient(mmHg) | 13.52±7.05 | 12.03±5.07 | 0.325 |
| ≥moderate pvl(n,%) | 1(2.38) | 8(18.18) | 0.030 |
| Post LVEF(%) | 62.00±8.92 | 57.50±9.37 | 0.038 |
| 2-year Clinical outcomes |  |  |  |
| All-cause mortality | 1(2.38) | 2(4.54) | ≥0.999 |
| Rehospitalization | 2(4.76) | 10(22.73) | 0.027 |
| Primary endpoint | 3(7.14) | 11(25.00) | 0.039 |

CV: calcium volume; BAV: bicuspid aortic valve ; PVL: paravalvular leak; LVEF: left ventricular ejection fraction

Supplementary table 2 Procedural and clinical outcomes according to excess leaflet CV in Type 1 BAV

| Type1 | No excess leaflet calcification(n=42) | Excess leaflet calcification(n=67) | P value |
| --- | --- | --- | --- |
| Procedural related outcomes |  |  |  |
| Conversion to open surgery(n,%) | 1(2.38) | 0 | 0.385 |
| Coronary obstruction(n,%) | 0 | 0 | - |
| Aortic root injury(n,%) | 0 | 0 | - |
| Implantation of two valves(n,%) | 3(7.14) | 6(8.96) | ≥0.999 |
| Stroke | 1(2.38) | 4(5.97) | 0.647 |
| Pacemaker | 4(9.52) | 12(17.91) | 0.276 |
| Echocardiographic findings |  |  |  |
| Mean transvalvular gradient(mmHg) | 11.72±7.00 | 9.73±4.58 | 0.098 |
| ≥moderate pvl(n,%) | 2(4.76) | 16(23.88) | 0.008 |
| LVEF(%) | 60.54±8.97 | 57.49±9.97 | 0.125 |
| 2-year Clinical outcomes |  |  |  |
| All-cause mortality | 2(4.76) | 4(5.97) | ≥0.999 |
| Rehospitalization | 4(9.52) | 18(26.87) | 0.030 |
| Primary endpoint | 4(9.52) | 19(28.36) | 0.028 |

Abbreviations as above.

Supplementary table 3 Procedural and clinical outcomes according to excess leaflet CV in TAV

| TAV | No excess leaflet calcification(n=84) | Excess leaflet calcification(n=65) | P value |
| --- | --- | --- | --- |
| Procedural related outcomes |  |  |  |
| Conversion to open surgery(n,%) | 2(23.81) | 1(1.54) | 0.558 |
| Coronary obstruction(n,%) | 1(1.90) | 0 | 0.385 |
| Aortic root injury(n,%) | 0 | 1(1.54) | ≥0.999 |
| Implantation of two valves(n,%) | 2(2.38) | 7(10.77) | 0.478 |
| Stroke | 0 | 3(4.62) | 0.283 |
| Pacemaker | 11(13.10) | 11(16.92) | 0.216 |
| Echocardiographic findings |  |  |  |
| Mean transvalvular gradient(mmHg) | 10.72±5.29 | 10.87±5.54 | 0.885 |
| ≥moderate pvl(n,%) | 3(3.57) | 7(10.77) | 0.104 |
| LVEF(%) | 60.69±8.06 | 58.58±9.63 | 0.162 |
| 2-year Clinical outcomes |  |  |  |
| All-cause mortality | 4(4.76) | 6(9.23) | ≥0.999 |
| Rehospitalization for HF | 6(7.14) | 7(10.77) | 0.547 |
| Primary endpoint | 7(8.33) | 10(15.38) | 0.807 |

Abbreviations as above
