# Supplementary material for: Clinical outcomes of bicuspid versus tricuspid aortic valve stenosis after transcatheter aortic valve replacement with self-expandable valves
Source: BMC Cardiovasc Disord. 2022 Dec 12;22:540. doi: 10.1186/s12872-022-02943-9 (PMC9743542; doi:10.1186/s12872-022-02943-9)

Supplementary Figure 1: Quantitative analysis of aortic valve calcification

Two regions are defined: aortic valve leaflet(from the basal annular plane to each cuspid tip(red bracket)); left ventricular outflow tract(from basal annular plane to 5mm underneath the left ventricle(yellow bracket))

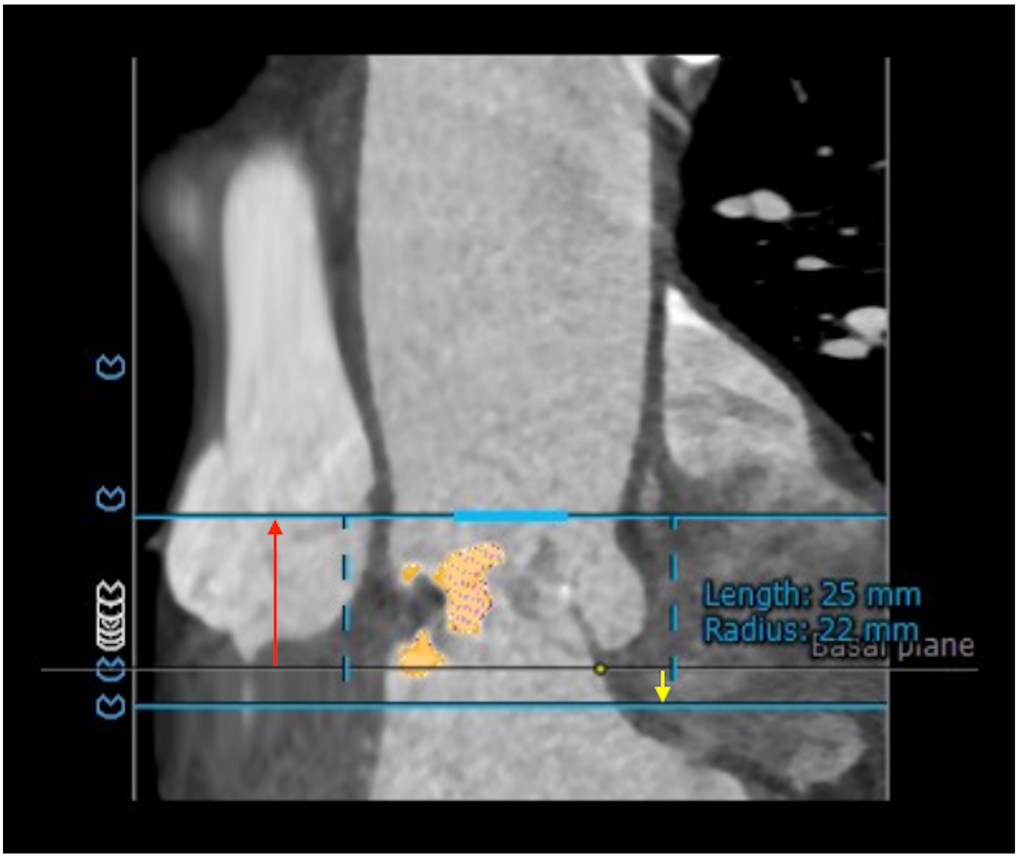

Supplement: Supplementary file 2 — Additional file 2: Supplementary Figure 1. Quantitative analysis of aortic valve calcification Two regions are defined: aortic valve leaflet(from the basal annular plane to each cuspid tip(red bracket)); left ventricular outflow tract(from basal annular plane to 5mm underneath the left ventricle(yellow bracket)). [file 12872_2022_2943_MOESM2_ESM.pdf]
