# Supplementary material for: Clinical outcomes of bicuspid versus tricuspid aortic valve stenosis after transcatheter aortic valve replacement with self-expandable valves
Source: BMC Cardiovasc Disord. 2022 Dec 12;22:540. doi: 10.1186/s12872-022-02943-9 (PMC9743542; doi:10.1186/s12872-022-02943-9)

Supplementary Figure 2 Comparisons of aortic valve calcium volume dichotomized by moderate/severe PVL

(A) Total aortic valve Leaflet CV; (B) Total LVOT CV

PVL: paravalvular leak; CV: calcification volume; LVOT: left ventricular outflow tract

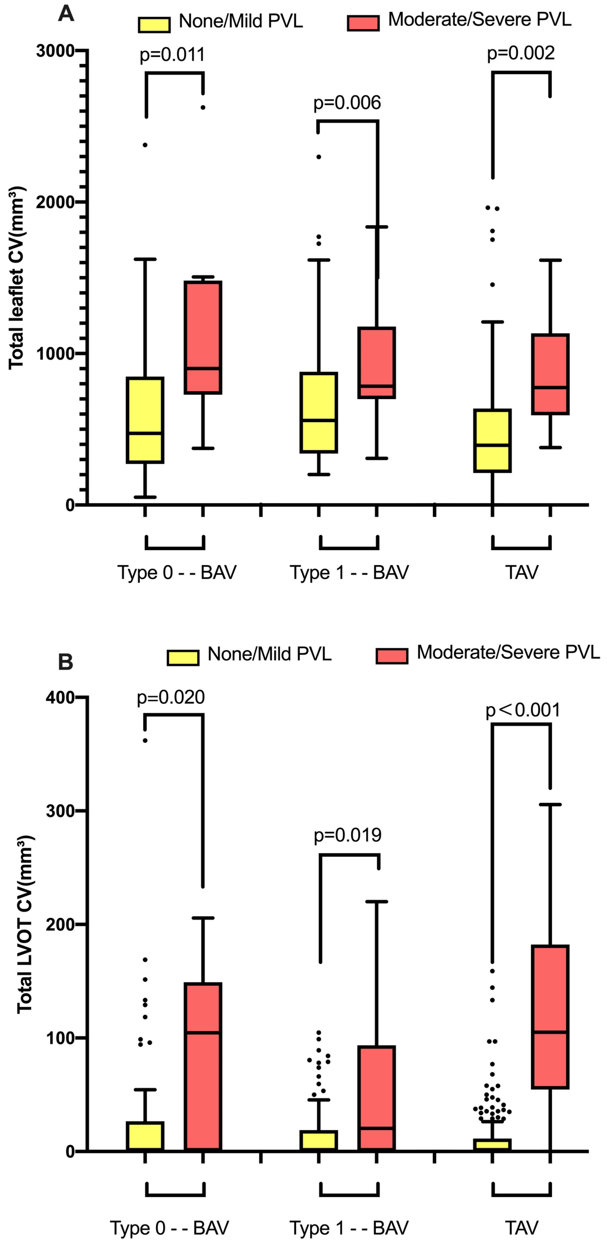

Supplement: Supplementary file 3 — Additional file 3: Supplementary Figure 2. Comparisons of aortic valve calcium volume dichotomized by moderate/severe PVL. (A) Total aortic valve Leaflet CV; (B) Total LVOT CV. [file 12872_2022_2943_MOESM3_ESM.pdf]
