# Supplementary material for: Clinical outcomes of bicuspid versus tricuspid aortic valve stenosis after transcatheter aortic valve replacement with self-expandable valves
Source: BMC Cardiovasc Disord. 2022 Dec 12;22:540. doi: 10.1186/s12872-022-02943-9 (PMC9743542; doi:10.1186/s12872-022-02943-9)

Supplementary Figure 3 Kaplan-Meier estimates of the rate of the primary composite endpoint in different aortic valve phenotype as varied by leaflet CV ≥ median among the specific group

(A) Type 0 BAV；(B) Type 1 BAV；(C) TAV

CV: calcification volume; BAV: bicuspid aortic valve; TAV: tricuspid aortic valve

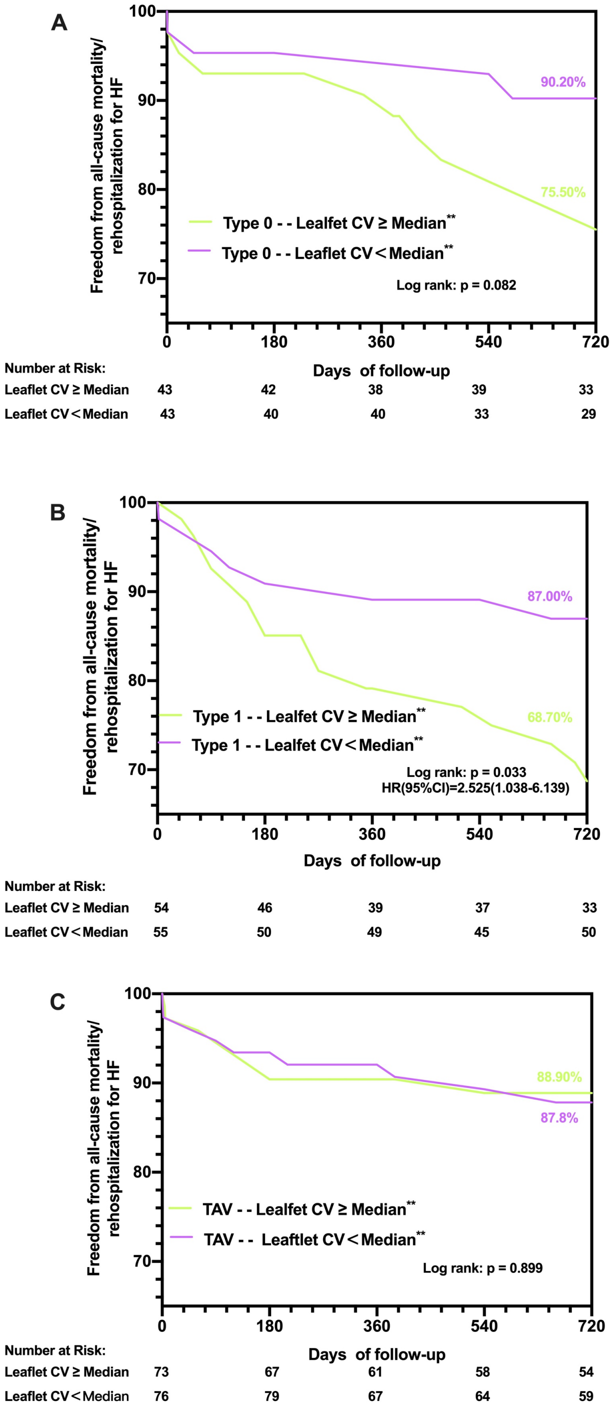

Supplement: Supplementary file 4 — Additional file 4: Supplementary Figure 3. Kaplan-Meier estimates of the rate of the primary composite endpoint in different aortic valve phenotype as varied by leaflet CV ≥ median among the specific group. (A) Type 0 BAV; (B) Type 1 BAV; (C) TAV. [file 12872_2022_2943_MOESM4_ESM.pdf]
